# The quality of the educational environment in a medical college in Saudi Arabia

**DOI:** 10.5116/ijme.58ce.55d2

**Published:** 2017-04-14

**Authors:** Abdullah H. Altemani, Tarig H. Merghani

**Affiliations:** 1 Family and Community Medicine Department, Faculty of Medicine, University of Tabuk, Saudi Arabia; 2 Department of Physiology, Faculty of Medicine, University of Tabuk, Saudi Arabia

**Keywords:** Dundee Ready Educational Environment Measure, students' perception of learning, students' perception of teachers, students' social self-perception, atmosphere, Saudi Arabia

## Abstract

**Objectives:**

The study aimed to examine the quality of the educational environment in the Faculty of Medicine, University of Tabuk, Saudi Arabia, and to compare between male and female students using the Dundee Ready Educational Environment Measure (DREEM).

**Methods:**

We utilized a cross-sectional survey design.  Participants were 221 medical students (96 males and 125 females) from all classes (1st to 6th year). Each participant responded to a translated version of the DREEM questionnaire that measures five domains: students' perception of learning (SPL), students' perception of teachers (SPT), students' academic self-perception (SASP), students' perception of atmosphere (SPA), and students' social self-perception (SSSP). Numerical differences between male and female students were analyzed using the Student's t-test.

**Results:**

The global average score of female students was significantly higher (105.0±22.9, 53% of maximum score) than male students (98.3±24.3, 49% of maximum score; t_(219)_= -2.119, p= 0.035). The major gender difference was found in the SPT domain, with a higher score in the females (60%) compared to the male (50%) cohort (t _(219)_= -5.519, p = 0.000). Differences in the other domains were statistically insignificant. Out of the 50 DREEM items, the items that need attention were 32 and 23 on the male and the female sides respectively.

**Conclusions:**

The perception of the educational environment by the female students is significantly better than male students. The study provides valuable information about many educational problems that need attention. DREEM-based surveys are highly recommended for periodic monitoring of the educational environment.

## Introduction

The term "educational environment" indicates all factors that affect the process of students' learning, such as the physical location, teachers, colleagues, and culture. It is defined as everything that happens within the classroom, department, faculty or university that is essential in determining the success of undergraduate medical education.^1^^,^^2^ The lecture room where students study, the way they interact with their teachers, the skills of their teachers in facilitating their learning, their attitudes to one another, and the culture in which they learn are all essential in determining success or failure of the learning process. The environment can be considered as a positive learning environment when it increases students' motivation towards learning, promotes their knowledge and skills, and stimulates their sense of social well-being. Evaluation of the educational environment has been regarded as a key to the delivery of high-quality medical education.^3^ The evaluation can be considered as a reflection of the quality of a curriculum and can be used to identify weak areas that require appropriate measures for improvement.^4^ The recent innovations in medical curricula and the increasing diversity of undergraduate students and medical courses have led to an increasing need to evaluate the educational environment of medical schools.^5^

The Dundee Ready Education Environment Measure (DREEM) is a validated tool that is commonly used for assessment of the educational environment created in the medical schools.^6^^-^^8^ Since its introduction in 1997,^6^ it has been used in many institutions all over the world to investigate an institutional status of the learning environment, to make comparisons between different groups within the same institution, and to find relations between students’ academic achievement and educational environment.^9^  Its translation from English into different languages and its use in many countries around the world indicates the international acceptance of this tool. The psychometric appraisal of the questionnaire has showed high levels of internal consistency and stability across different settings.^10^^,^^11^ Questions about basic psychometric properties and construct validity have nevertheless been raised.^12^^,^^13^ It has been suggested that removal of certain repeated items might improve the goodness of fit of the five-factor structure of the questionnaire.^11^^-^^13^

The DREEM may be useful for educators to make national and international comparisons between medical schools, with the purpose of identifying areas of concern that can be improved in the future.^8^^,^^13^ Previous studies in the Middle East were conducted to evaluate students’ perceptions within and between institutions; to explore gender discrepancies; and to compare medical schools adopting contrasting educational strategies.^14^^-^^17^ The reported scores agree with the general conclusion that DREEM scores in traditional schools are generally less than 120.^9^ Similar results in Europe showed the existence of several educational problems associated with the traditional curricula and the teaching methodologies.^4^ In a recent systematic review, it has been concluded that the DREEM inventory is the most suitable tool for measuring the educational environment for undergraduate medical students.^18^ It is also useful in guiding remedial measures.^19^ Repeated reassessments would reflect the pattern of change overtime and show the effectiveness of the corrective actions.^20^

The Faculty of Medicine, University of Tabuk, is a newly established medical college with a history of about nine years. It follows a hybrid modular curriculum that adopts both traditional and problem-based learning (PBL) for teaching undergraduate students. The Faculty includes two separate sections, one for the male students and another for the females, both of which are related to the University. Although they share the same contents of the curriculum, the teachers of each section and the lecture rooms are different. It is unknown whether perceptions of the students towards their educational environments in the two sections are the same. Therefore, the aim of this study was to examine qualities of the educational environments in the male and the female sections of the Faculty by exploring areas of strength and weakness, as perceived by the medical students using the DREEM tool.

## Methods

### Study design

Undergraduate students of the Faculty of Medicine, University of Tabuk, Saudi Arabia, were all invited to participate in this cross-sectional study.

### Participants

The study was carried out with 221 medical students from all classes (1st to 6th year). Approval and ethical clearance were obtained from the Research Committee of the Faculty of Medicine, University of Tabuk. The completion of the anonymous DREEM questionnaire was undertaken on a voluntary basis. All data was handled in accordance with the ethical principles of medical research developed by the World Medical Association of Helsinki.^21^

**Table 1:** A comparative analysis in the subscale DREEM scores between males and females (n= 221)

DREEM domain (Ideal mean score)	Campus M (96) F (125)	Mean n (%)	SD	t_(df)_, p value
Students' perception of learning (48)	Male	23.5(49)	6.4	t_(219)_= -1.416, p= 0.158
	Female	24.7(51)	5.9	
Students' perception of teachers (44)	Male	22.0(50)	6.7	t_(219)_= -5.519, p= 0.000^*^
	Female	26.6(60)	5.9	
Students' academic self-perception (32)	Male	15.4(48)	5.5	t_(219)_= .644, p= 0.520
	Female	14.9(47)	5.3	
Students' perception of atmosphere (48)	Male	22.1(46)	7.9	t_(219)_= -1.522, p= 0.129
	Female	23.7(49)	7.9	
Students social self-perception (28)	Male	15.4(55)	4.1	t_(219)_= .422, p= 0.673
	Female	15.2(54)	3.8	
Overall maximum mean score (200)	Male	98.3(49)	24.3	t_(219)_= -2.119, p= 0.035^*^
	Female	105.0(53)	22.9	

### Instrument

The Arabic translation of the 50-item DREEM questionnaire was used to measure students’ perceptions of the educational environment in this study.^14^^-^^17^  It is a 50-statement, closed-ended questionnaire that requests information about five domains: students’ perception of learning (SPL, 12 statements), students’ perception of teachers (SPT, 11 statements), students’ academic self-perception (SASP, 8 statements), students’ perception of atmosphere (SPA, 12 statements), and students’ social self-perception (SSSP, 7 statements). Each statement response scored 0-4 on a 5-point Likert-type scale (strongly disagree, disagree, uncertain, agree, and strongly agree). The negative statements (questions number 4, 8, 9, 17, 25, 35, 39, 48, and 50) were scored in reverse. A very high score indicates excellent performance.  The mean total score for each subscale can be calculated. The mean maximum score that can be achieved by an ideal educational environment is 200. The data can be expressed as percentages of maximum scores in the respective subscale or of the global scale. The individual items of the DREEM were analyzed according to the score of each item.^7^ A mean score of 3 or more indicates a strong item (positive), a mean score of 2 or less indicates that the item needs attention (negative), and a mean score between 2 and 3 indicates that the item needs improvement.

### Sample size and data collection

All students were approached. A total of 221 students (96 males and 125 females) participated in the study with a total response rate of 69%. The study was explained to the participants before distribution of the questionnaires. The participants received clear information about the voluntary participation and the anonymity of the process. All the participants completed the questionnaires within the same day of distribution. Non-teaching collaborators facilitated the process and collected the completed questionnaires.

**Table 2:** Students’ Perception of Learning (n= 221)

Item		Male n= 96		Female n=125		t (df), p value
		Mean (SD)	Analysis	Mean (SD)	Analysis	
1.	I am encouraged to participate during teaching sessions	2.2 (1.1)	Needs improvement	2.3 (1.0)	Needs improvement	-0.769_(219)_, 0.443
7.	The teaching is often stimulating	1.9 (1.2)	Needs attention	2.0 (1.1)	Needs attention	-0.897_(219)_, 0.371
13.	The teaching is student-centered	2.2 (1.4)	Needs improvement	2.3 (1.1)	Needs improvement	-0.595_(219)_, 0.553
16.	The teaching helps to develop my competence	1.9 (1.1)	Needs attention	1.8 (1.2)	Needs attention	0.489_(219)_, 0.625
20.	The teaching is well-focused	2.2 (1.1)	Needs improvement	2.4 (1.2)	Needs improvement	-1.165_(219)_, 0.245
22.	The teaching helps to develop my confidence	1.8 (1.1)	Needs attention	1.8 (1.2)	Needs attention	-0.170_(219)_, 0.865
24.	The teaching time is put to good use	1.7 (1.1)	Needs attention	2.0 (1.3)	Needs attention	-1.787_(219)_, 0.075
25.	The teaching over-emphasizes factual learning	2.0 (1.1)	Needs attention	2.0 (1.2)	Needs attention	0.171_(219)_, 0.865
38.	I’m clear about the learning objectives of the course	1.7 (1.2)	Needs attention	1.9 (1.1)	Needs attention	-0.873_(219)_, 0.384
44.	The teaching encourages me to be an active learner	1.6 (1.2)	Needs attention	2.0 (1.2)	Needs attention	-2.218 _(219)_, 0.028^*^
47.	Long-term learning is emphasized over short-term	2.7 (1.3)	Needs improvement	3.0 (1.2)	Strong	-1.636 _(219)_, 0.103
48.	The teaching is too teacher-centered	1.5 (1.3)	Needs attention	1.1 (1.1)	Needs attention	2.158 _(219)_, 0.032^*^

### Data analysis

The collected data was manually entered into a Microsoft Excel data sheet (version 2010) and then into the Statistical Package for the Social Sciences (SPSS Inc., Chicago, IL. Version 20) for data analysis. The variables were described using means and standard deviations (SD) and percentages of maximum scores. The numerical differences between the males and the females were analyzed with the independent student's t-test. P values < 0.05 were considered statistically significant.

**Table 3:** Students’ Perception of Teachers (n= 221)

Item		Male n= 96		Female n= 125		t (df), p value
		Mean (SD)	Analysis	Mean (SD)	Analysis	
2.	The teachers are knowledgeable	2.4 (1.1)	Needs improvement	2.6 (1.0)	Needs improvement	-1.720 _(219)_, 0.087
6.	The teachers adopt a patient-centered approach to consulting	2.0 (1.3)	Needs attention	2.1 (1.5)	Needs improvement	-0.700 _(219)_, 0.485
8.	The teachers ridicule the students	2.0 (1.3)	Needs attention	2.6 (1.1)	Needs improvement	-3.556 _(219)_, 0.000^*^
9.	The teachers are authoritarian	1.5 (1.2)	Needs attention	2.3 (1.2)	Needs improvement	-4.968 _(219)_, 0.000^*^
18.	The teachers have good communication skills with patients	1.9 (1.2)	Needs attention	2.1 (1.3)	Needs improvement	-1.380 _(219)_, 0.169
29.	The teachers are good at providing feedback to students	1.8 (1.2)	Needs attention	2.2 (1.1)	Needs improvement	-3.038 _(219)_, 0.003^*^
32.	The teachers provide constructive criticism here	1.5 (1.4)	Needs attention	2.2 (1.2)	Needs improvement	-3.748 _(219)_, 0.000^*^
37.	The teachers give clear examples	2.4 (1.1)	Needs improvement	2.7 (0.9)	Needs improvement	-2.359 _(219)_, 0.019^*^
39.	The teachers get angry in teaching	1.8 (1.3)	Needs attention	2.5 (1.2)	Needs improvement	-3.878 _(219)_, 0.000^*^
40.	The teachers are well-prepared for their teaching sessions	2.1 (1.3)	Needs improvement	2.5 (1.1)	Needs improvement	-2.857 _(219)_, 0.005^*^
50.	The students irritate the teachers	2.7 (1.3)	Needs improvement	2.8 (1.2)	Needs improvement	-0.422 _(219)_, 0.673

## Results

Table 1 shows that the overall mean DREEM score of the female students was found to be significantly higher (105.0±22.9, 53% of maximum score) than that of male students (98.3±24.3, 49% of maximum score; t_(__219)_=-2.119, p=0.035). The major difference was found in the SPT domain, with a higher score on the female side (60%) compared to that of the males (50%; t_(__219)_=-5.519, p=0.000).    Table 2 shows the results of SPL. A total of eight out of twelve items need attention (mean score ≤ 2.0) in each of the two sides (male and female). The strongest item (mean score ≥ 3.0) was the item number 47 (Long-term learning is emphasized over short-term) for the females, whereas that item needs improvement for males.  Significant gender differences were found in the item number 44 (The teaching encourages me to be an active learner; t_(__219)_=-2.218, p=0.028), and the item number 48 (The teaching is too teacher-centered; (t_(219)_=2.158, p=0.032).

Table 3 shows a significant gender discrepancy in the SPT domain. Seven out of eleven items need attention in the male side compared to none in the other side. Significant statistical differences were found in item number 8 (The teachers ridicule the students; t_(219)_=-3.556, p=0.000), number 9 (The teachers are authoritarian; t_(219)_=-4.968, p=0.000), number 29 (The teachers are good at providing feedback to students; t_(219)_=-3.038, p=0.003), number 32 (The teachers provide constructive criticism here; t_(219)_= -3.748, p=0.000), number 37 (The teachers give clear examples; t_(219)_=-2.359, p=0.019), number 39 (The teachers get angry in teaching; t_(219)_=-3.878, p=0.000), and number 40 (The teachers are well-prepared for their teaching sessions; t_(219)_=-2.857, p=0.005).

Analysis of the SSSP domain showed three items that need attention in each of the two sections (Table 4). Strong items were the item number 15 in the two sections (I have good friends in this course; t_(__219)_=1.888, p=0.060) and number 46 in the female section (My accommodation is pleasant; t_(219)_=-1.761, p=0.080). The lowest score, mean (SD) = 0.8 (1.1), was observed in the male section for the item number 3 (There is a good support system for students who get stressed; t_(__219)_=-3.199, p=0.002 ).

**Table 4:** Students’ Social Self-Perception (n= 221)

Item		Male n= 96		Female n= 125		t test (df), p value
		Mean (SD)	Analysis	Mean (SD)	Analysis	
3.	There is a good support system for students who get stressed	0.8 (1.1)	Needs attention	1.3 (1.1)	Needs attention	-3.199 _(219)_, 0.002*
4.	I am too tired to enjoy the course	1.3 (1.3)	Needs attention	1.2 (1.2)	Needs attention	0.497 _(219)_, 0.620
14.	I am rarely bored in this course	2.0 (1.3)	Needs attention	1.7 (1.4)	Needs attention	1.603 _(219)_, 0.110
15.	I have good friends in this course	3.3 (1.0)	Strong	3.1 (1.2)	Strong	1.888 _(219)_, 0.060
19.	My social life is good	2.8 (1.2)	Needs improvement	2.5 (1.3)	Needs improvement	1.698 _(219)_, 0.091
28.	I seldom feel lonely	2.4 (1.3)	Needs improvement	2.3 (1.2)	Needs improvement	0.242 _(219)_, 0.809
46.	My accommodation is pleasant	2.9 (1.4)	Needs improvement	3.2 (1.1)	Strong	-1.761 _(219)_, 0.080

## Discussion

The DREEM inventory is regarded as the most suitable instrument for measuring the educational environment in undergraduate medical institutions.^18^ In this study, we used the DREEM for evaluating the educational environment in the Faculty of Medicine, University of Tabuk. Our results showed that many educational issues need attention and re-evaluation. Our scores agree with the results of several studies conducted in other Saudi universities. Examples include King Abdul Aziz University (102.0),^14^ King Khalid University (112.95),^15^ and Qassim University (112.0).^16^ It is worth noting that the score of Taibah University was 109/200 in the year 2007/2008, and then after implementation of remedial measures, it reached 120.7/200 in the year 2010/2011.20 Many of the issues raised by the students in these universities were similar to ours, and therefore our future management plans have to be organized in the light of the reforms taken by these institutions. Our scores, being less than 120, indicate the dominance of the traditional methods of teaching in our college.^9^ Reassessment using DREEM could be of benefit in measuring the change following implementation of corrective measures.^20^

One important application of the DREEM inventory is the analysis of the individual items. This directly shows the strengths and the weaknesses of different aspects of the educational environment. Our results showed many weak issues raised by the students that should receive adequate attention. The majority of the items in the domain of learning scored 2.0 or less indicating problems. The total average for this domain was 49% for the males and 51% for females. The item with the lowest score was number 48 (The teaching is too teacher-centered). In the teacher-centered education, the teacher directs the lecture-room activities and ensures that the students will not miss an important topic; however, it does not allow them to communicate, express themselves, and direct their learning. Negative perceptions of the learning subscale were also reported in Spain, and it was attributed to the use of traditional methods of teaching.^4^ Greater efforts are needed to introduce learning activities that allow the students to interact with each other and to work in groups to develop their communication skills and collaborative behavior. The teachers should be encouraged to enhance their capabilities and to introduce innovative strategies to combat the negative perceptions of their students. On the other hand, item number 47 (Long-term learning is emphasized over short-term) is perceived positively, especially by the females. It is worth noting that the durable knowledge is one of the primary goals of medical education.

The total DREEM score was higher for females compared to males. Similar findings were reported in many other Saudi and non-Saudi universities.^20^^,^^22^ A significant gender discrepancy exists in the students’ perceptions of teachers (SPT) domain. Female students scored a significantly higher value than their male counterparts. The most important items that need attention were number 9 (The teachers are authoritarian) and 32 (The teachers provide constructive criticism here) indicating a need for evaluation of teachers' attitude and behavior. Informing the teachers about these results could be of benefit.

The domain of students' social self-perception (SSSP) showed more positive than negative scores in both the male (55%) and the female (54%) sections. Items asking about friends (number 15) and accommodation (number 46) got the highest scores. Similar results were reported in both national and foreign universities.^4^^,^^16^ The presence of friendly relationships between the students reflects a healthy environment. The nature of the PBL sessions that encourage interaction between the students was mentioned as an explanation in a previous study.^16^ Item number 3 that asks about “presence of a good support system for stressed students” got the lowest score among all the 50 items. This indicates a grave lack of supportive strategies for stressed students. Unfortunately, this is prevailing in many other Saudi Universities.^16^^,^^17^ A recent study reported that the top stressors for undergraduate students are fear of failing a course, examination results, and concerns regarding completion of clinical work.^23^ Stress may affect students’ well-being and impacts their academic performance. It is recommended that the college, during academic advising sessions, should offer consultation and teach the students how to manage and cope with the various stressful situations, whether academic, social or financial.

### Limitations

The study has many limitations that need to be considered for interpretation. First, the sampling method depended on the voluntary participation of participants and therefore sampling bias are expected since the distribution across the year levels were not equal. Second, the nature of self-reporting questionnaires is likely to be associated with response bias.

## Conclusions

In conclusion, our results showed many problems in the educational environment, with a higher DREEM score in the female section compared to that of males. Greater efforts are needed to manage the negative items that include the negative perception of teaching, the stressful environment, and the lack of supportive strategies for the stressed students. The study showed that monitoring of the educational environment could provide valuable information that could be used by the educators to address the issues that need attention and to implement the necessary changes for improvement. Further studies are needed to find the causes of stress among the students and to evaluate the degree of improvement over time.

## Conflict of Interest

The authors declare that they have no conflict of interest.
